# Aronia Berry Processing by Spray Drying: From Byproduct to High Quality Functional Powder

**DOI:** 10.17113/ftb.57.04.19.6369

**Published:** 2019-12

**Authors:** Senka Vidović, Milica Ramić, Rita Ambrus, Jelena Vladić, Piroska Szabó-Révész, Aleksandra Gavarić

**Affiliations:** 1 Faculty of Technology, University of Novi Sad, Bulevar cara Lazara 1, 21000 Novi Sad, Serbia; 2 Department of Pharmaceutical Technology, Eotvos 6, University of Szeged, 6720 Szeged, Hungary

**Keywords:** aronia, by-product, ultrasound assisted extraction, spray drying, powder

## Abstract

The main aim of this study is to analyze the solid-liquid extraction followed by spray drying as a technological pathway for utilization of aronia fruit dust, a byproduct of filter tea factory. In the current study, ultrasound-assisted extraction was applied for the production of aronia liquid feed and maltodextrin was used as a carrier and encapsulating agent. In spray drying, the influence of inlet temperature and maltodextrin type and mass fraction on process efficiency and powder properties were observed. The physical and chemical properties of the obtained powders were characterized. It was determined that the powder produced using inlet temperature 140 °C and 40% maltodextrin with dextrose equivalent (DE) 19.7 had the most desirable characteristics. It was observed that the increase in maltodextrin mass fraction decreases the powder moisture content, hygroscopicity and the content of bioactive compounds, but increases water solubility index and particle size. The increase in dextrose equivalent of maltodextrin increases the powder hygroscopicity and water solubility index, while the increase of inlet temperature causes a decrease in moisture content of aronia powders.

## INTRODUCTION

In recent years, *Aronia melanocarpa* L. (also known as aronia or black chokeberry) has attracted a great interest due to its unique composition of bioactive compounds, which ensures multiple health-promoting properties (*1*). Namely, it was found that aronia is one of the richest sources of phenolic compounds and that the content of proanthocyanidins, anthocyanins and phenolic acids in this fruit is quite high (*2*). Polymeric procyanidins with up to 66% of total polyphenols are the major subclass and the main contributors to the specific astringent, tart taste of aronia berry and its products (*3*). Anthocyanins make approx. 25% of its total phenolic compounds. They consist mainly of cyanidin glycosides: glucoside, galactoside, arabinoside and xyloside, with cyanidin arabinoside and galactoside accounting for 60–75% of the total anthocyanins (*3*). According to the study of Szopa *et al*. (*4*), aronia berry contains significant amounts of two phenolic acids: chlorogenic and neochlorogenic acid. Antioxidant activity of aronia is significantly high (*5*). Positive effects of aronia on human health have been the subject of numerous studies. A few of them showed favourable effects of aronia in the control and prevention of diabetes and diabetes-associated complications (*6*), prevention and treatment of cardiovascular diseases, treatment of colon cancer and hepatoprotective effect (*1*, *2*, *7*).

In food industry, various byproducts or wastes are generated during the processing of aronia berries. Aronia cake is byproduct/waste generated during the production of aronia juice. After juice production, by application of adequate technological treatment, this cake is transformed into the form that is further used in the production of granola, chocolate, natural food additives, *etc*. For its application in fruit filter tea production, according to Ramić *et al*. (*8*), such cake needs to be dried, milled, ground and fractionated. After such processing, approx. 20% of totally processed cake is of particle size smaller than the size of pores of filter tea bags, and as such it is discharged as byproduct from filter tea factory. Since this fraction cannot be packed into filter bags, it represents a byproduct also known as ‘aronia fruit dust’.

According to our previous study (*8*), aronia fruit dust, a byproduct/waste of aronia berry fruit processing in food factory, represents a valuable source of polyphenolic constituents. The same study showed that ultrasound-assisted extraction can be successfully applied for recovery of aronia polyphenolic constituents and for production of new functional products in form of liquid extracts. However, to produce a more stable products that are more easily handled, adequate drying technique, intended for transformation of liquid extracts into the powder form, should be used.

The production of stable powders, especially powders made from liquid extracts with high concentration of low-molecular-mass sugars such as aronia juice and its liquid extracts, represents one of the major challenges in the powder production. Several drying techniques have been proposed for the production of herbal and fruit powders, among them spray drying has been most widely utilized. According to Gonnissen *et al.* (*9*), continuous technological operations for production of powders, such as spray drying, have been preferred over batch processes due to reduced time to market, scale-up benefits and improved quality of product (absence of batch to batch variations). For production of adequate powder, spray drying technology requires adequate adjustments of operating conditions and composition of the feed solution (mixture of liquid extract and additives). Adequate adjustments of operating conditions refer to alteration of inlet (*t*_i_) and outlet (*t*_o_) temperatures, atomization flow, liquid flow rate, concentration of solids in liquid feed, and concentration of added drying additives.

Many problems can occur during powder production and further storage and powder utilization. One of the most frequent problems encountered during spray drying is certainly the stickiness of extracts on the drying unit walls (*10*). During storage, the stickiness issue can also occur, and this affects the product handling. The stickiness (cohesion and adhesion) in the process of drying is caused by the higher content of low-molecular-mass sugars present in the liquid feed and/or by application of higher drying temperature than glass transition temperature (*t*_g_) of the liquid feed. Low-molecular-mass sugars have low *t*_g_, therefore, if their concentration in liquid feed is high, they can induce the decrease of *t*_g_ of the entire feed. According to Johari *et al*. (*11*), if *t*_g_ of the feed is lower than inlet tempreture (*t*_i_) of the drying process (for at least 10–20 °C), the process will be unsuccessful. Since water has very low *t*_g_ (-135 °C), it can provoke significant *t*_g_ depression of produced powders causing a decrease of the free-flowing properties, an increase of caking property, as well as manipulating problems during further use of such products. Therefore, considering the water content in powders, two main requirements should be respected: production of powders with the lowest possible content of water, and their further adequate packaging and storage in terms of adequate moisture and temperature conditions.

Liquid extracts can be successfully dried with improvements in dryer design/operation, previous addition of carriers to extracts, or both (*10*). Analysis of the main influential parameters and further adequate set-up of drying conditions can achieve improvement of the drying operation. Therefore, to ensure the production of quality powders from liquid feeds with high sugar content, such as aronia liquid extracts, adequate set-up of *t*_i_ and outlet temperature (*t*_o_), and addition of adequate drying carrier need to be included. Currently, maltodextrins (MDs) are considered as one of the most suitable drying carriers in the food and pharmaceutical industries (*12*-*14*).

Therefore, the main aim of this study is to analyze and propose a new technological pathway for utilization of aronia byproducts/wastes. Hereof, solid-liquid extraction followed by spray drying was applied for the recovery of bioactive compounds from aronia fruit dust and for the production of new stable functional product in powder form. Drying was performed after the addition of MDs as carrier and encapsulating agents. The impact of different MDs and their mass fraction, as well as of *t*_i_, on process efficiency and powder properties (chemical and physical) was observed.

## MATERIALS AND METHODS

### Samples and reagent

Folin-Ciocalteu reagent and gallic acid were purchased from Sigma-Aldrich Chem, Merck (Steinheim, Germany). Maltodextrins classified by dextrose equivalent (DE) as DE 19.7, DE 13.1 and DE 5.9 were purchased from Brenntag (Mülheim, Germany). All other chemicals and reagents were of analytical reagent grade. Aronia fruit dust was donated by Fructus (Bačka Palanka, Serbia), a producer of herbal and fruit filter tea.

### Preparation of liquid feed

For the production of aronia liquid extract, ultrasound-assisted extraction was applied. Extraction conditions were set according to the previously defined optimal conditions: temperature 70 °C, extraction time 80.1 min, ultrasonic power 206.64 W and 50% (*m*/*m*) ethanol as extraction solvent. In all experimental runs, 10.0 g of fruit dust were mixed with 50 mL of solvent in 100-mL flasks. Ultrasound-assisted extraction was performed in sonication water bath (2510, Branson, Danbury, CT, USA) with fixed frequency at 40 kHz (*10*). MDs of different dextrose equivalents (DE 19.7, 13.1 and 5.9) at different mass fractions (20, 40 and 60%) were added to the prepared liquid extracts and mixed for 0.5 h prior to spray drying. The described procedure was likewise repeated for the preparation of 9 different liquid feeds containing maltodextrins DE 19.7, 13.1 and 5.9 at mass fractions 20, 40 and 60%.

### Spray drying process

The prepared liquid feeds were dried using laboratory mini spray dryer B-290 (Büchi, Flawil, Switzerland) with following characteristics: evaporation rate 1.0 L/h for water solutions, maximal inlet temperature (*t*_i_) 230 °C, heating capacity 230 W, maximal air flow 35 m^3^/h, nozzle diameter 0.7 mm. Liquid feeds were dried at three different inlet temperatures *t*_i_=120, 140 and 160 °C, while outlet temperature was kept constant at *t*_o_=(80±5) °C. The feeding rate was constant at 10 mL/min. The obtained powder was separated from the air by a cyclone. The production yield of spray drying was determined according to the mass of total solids measured in the feed and the mass of dry powder obtained at the end of the process in the powder receptacle. Yield was expressed as a percentage of the mass of the final product compared to the total amount of the spray-dried materials.

### Powder characterization

#### Moisture content

The moisture analyzer with halogen heating (MA 35; Sartorius, Göttingen, Germany) was used to determine the moisture content at set temperature of 105 °C (laboratory oven series 11446; Sutjeska, Belgrade, Serbia). Moisture content analyses of all samples were performed immediately after the spray drying. All experiments were performed in three replicates.

#### Hygroscopicity

Hygroscopicity of aronia powders was analyzed according to the procedure described previously by Vladić *et al.* (*15*). Powder sample, approx. 1 g, was placed in an airtight container at 25 °C or in a desiccator filled with NaCl-saturated solution (relative humidity 70%). Increase in mass, caused by absorbed water, was measured after 48 h. Hygroscopicity was expressed in g of absorbed water per 100 g of powder. All experiments were performed in three replicates.

#### Water solubility index and water absorption index

The water solubility index (WSI) is used as a measure of degradation of powder constituents. The low WSI represents a minor degradation of constituents, defining a low number of soluble molecules present in the powder. Water absorption index (WAI) is an indicator of the powder ability to absorb water. WAI depends on the availability of hydrophilic groups, responsible for binding water molecules, and on the gel-forming capacity of macromolecules (*16*). The low WAI indicates better stability during storage.

The WSI and WAI were determined according to the procedure previously described by Phoungchandan and Sertwasana (*17*). A mass of 1.25 g powder and 15 mL water were vigorously mixed in a 50-mL centrifuge tube. Afterwards, the mixture was incubated in a water bath at 30 °C for 30 min, and centrifuged for 15 min at 3000×*g* (Tehnica LC-320; Železniki, Slovenia). The supernatant was collected in a pre-weighed Petri dish and the residue was weighed after being oven dried at 105 °C overnight. The amount of solids in the dried supernatant was calculated as a percentage of the total dry solids in the original 1.25 g of sample, and is an indicator of WSI. The WAI was calculated as the mass of solid pellets remaining after centrifugation divided by the mass of the original dry sample. All experiments were performed in three replicates.

#### Wettability

For analysis of product/powder wettability, the OCA - optical contact angle system (OCA 20; DataPhysics Instruments, GmbH, Filderstadt, Germany) was applied. In the analysis by a Specac hydraulic press (Specac Inc., Fort Washington, PA, USA), 0.15 g aronia powder was compressed under the pressure of 1 t. The wetting angles were determined after 4.3 μL of distilled water was dropped onto the surface of the pressings. Using the circle fitting method of the OCA system, the change in the wetting angle was registered, from 1 to 25 s (a minimum of 5 parallel numbers).

Two liquids with known polar (*γ*_l_^p^) and dispersion (*γ*_l_^d^) liquid surface tensions were used for the measurement. In our study these were bidistilled water (*γ*_l_^p^=50.2 mN/m, *γ*_l_^d^=22.6 mN/m) and diiodomethane (*γ*_l_^p^=1.8 mN/m, *γ*_l_^d^=49 mN/m). According to Wu and Brzozowski (*18*), the solid surface free energy was calculated using the following equation:

 /1/

where *Θ* is the contact angle, *γ*_s_ is the solid surface free energy and *γ*_l_ is the liquid surface tension. The percentage polarity can be calculated from the *γ*^p^ and *γ* values (*18*):

/2/

Analysis of powder morphology by scanning electron microscopy

The morphology of the obtained aronia powder particles was analyzed similarly to our previous study (*15*) using scanning electron microscopy (SEM; Hitachi S4700; Hitachi Scientific Ltd., Tokyo, Japan). To induce electric conductivity on the surface of the samples, sputter coating apparatus (Bio-Rad SC 502; VG Microtech, Uckfield, UK) was applied. The air pressure was 1.3-13.0 mPa.

#### Analysis of powder particle size using microscopic measurement

For measurement of powder particle size, optical microscope LEICA Image Processing and Analysis System (LEICA Q500MC; LEICA Microsystems Cambridge Ltd., Cambridge, UK) was used. Dry powder was dispersed on the slide and 40-100× magnifications were applied. The particles were described by their length and width. Description was made based on the measurement of 350 particles per sample.

#### Differential scanning calorimetry

The differential scanning calorimetry (DSC) measurements were performed similar to our previous study (*17*) using Mettler Toledo DSC 821e thermal analysis system, with STAR^e^ thermal analysis program v. 6.0 (*19*). A mass of 2–5 mg of powder was analyzed in the temperature range from 25 to 300 °C. Applied heating rate was 5 °C/min, while argon, at flow rate of 10 L/h, was used as a carrier gas.

#### X-ray powder diffraction

X-ray powder diffraction (XRPD) spectra were recorded similar to our previous study (*15*) using BRUKER D8 Advance X-ray diffractometer system (Bruker AXS GmbH, Karlsruhe, Germany) with Cu Kα1 radiation (*λ*=1.5406 Å) over the interval 2*Θ*=5-30°. The applied analysis conditions were as follows: target Cu, filter Ni, voltage 40 kV, current 40 mA, time constant 0.1 s and angular step 0.010. In the determination of the degree of crystallinity, the total area of the three peaks with the largest intensity was examined, after smoothing and background removal.

### Content of bioactive compounds

#### Analysis of total phenolic content

In the obtained aronia powders, the total phenolic content (TPC) was determined by standard Folin–Ciocalteu procedure (*20*). TPC was expressed in mg of gallic acid equivalent per g of obtained powder (mg GAE/g). All experiments were performed in three replicates.

#### Analysis of monomeric anthocyanin content

In the obtained aronia powders, the monomeric anthocyanin content was estimated using spectrophotometer (6305 UV-VIS; Cole-Parmer Ltd. (Jenway), Stone, UK) and the pH differential method reported by Abu Bakar *et al*. (*21*) with minor modifications (*22*). Two buffer systems, potassium chloride buffer, pH=1.0 (0.0025 M) and sodium acetate buffer, pH=4.5 (0.4 M), were used. Briefly, 400 μL of sample (diluted liquid extract) were added to 3.6 mL of corresponding buffer solutions and absorbance was measured against a blank probe at 510 and 700 nm:

Δ*A*=(*A*_510 nm_-*A*_700 nm_)_pH=1.0_ - (*A*_510 nm_-*A*_700 nm_)_pH=4.5_ /3/

Anthocyanin content in the extract was calculated and expressed as cyanidin-3-glycoside equivalent (C3G):

*w*(MA) *=*(*A*·*M*·DF·1000)/*ε* /4/

where Δ*A* is the difference in the absorbance, *M* is the molecular mass of cyanidin-3-glucoside (449.2 g/mol), DF is the dilution factor of the samples and *ε* is the molar absorption coefficient of cyanidin-3-glucoside (26.900 M^-1^·cm^-1^). Results were expressed in mg of cyanidin-3-glucoside equivalents per g of obtained powder (mg C3G per g). All experiments were performed in three replicates.

### Statistical analysis

The experiments were carried out in triplicate and the results were expressed as mean value±standard deviation and considered significantly different when p≤0.05. One-way ANOVA was conducted to test the influence of individual factors on the observed property and Tukey's HSD *post hoc* test was used to determine differences between the mean values (STATISTICA v. 8) (*23*).

## RESULTS AND DISCUSSION

This study explored the possibility of the use of aronia fruit dust with the application of solid-liquid extraction followed by spray drying. Research focused on the influence of two dominant process parameters in spray drying: drying and encapsulating agents (type and mass fraction) and *t*_i_ (at constant *t*_o_). The effect of drying agents was investigated in experiments where three different maltodextrins (MDs: DE 5.9, 13.1 and 19.7) in three different mass fractions (20, 40 and 60%) were applied in spray drying of aronia liquid extract (*t*_i_ and *t*_o_ were set at 120 and 80 °C) (Table 1). Effects of *t*_i_ on the process efficiency, properties and quality of the obtained powders were studied at three different temperatures: 120, 140 and 160 °C, with MD DE 19.7 (*t*_o_=80 °C). This MD was chosen as drying agent because it is the most common MD in the food industry. Results of this part of research are presented in Table 2. During the investigation, 15 different powders were prepared.

**Table 1:** Impact of maltodextrin (MD) type and mass fraction on physical and chemical properties of aronia powders obtained at inlet and outlet temperatures of 120 and 80 °C, respectively

DE of MD	*w*(MD)/%	Process efficiency/%	*w*(moisture)/%	Hygroscopicity/%	WAI (*m*(solid)_centrifugate_/*m*(dry sample))/(g/100 g)	WSI (*m*(solid)_supernatant_/*m*(solid)_sample_)/%	*w*(TPC as GAE)/(mg/g)	*w*(TMA as C3G)/(mg/g)
5.9	20	64.00	(4.5±0.1)^bc^	(13.9±0.1)^abcd^	(0.41±0.01)^a^	(47.1±0.7)^g^	(277.0±0.4)^ab^	(32.1±0.7)^bc^
5.9	40	72.8	(4.6±0.2)^bc^	(13.1±0.6)^bcd^	(0.40±0.01)^ab^	(49.7±0.2)^f^	(238.3±0.5)^e^	(29.8±0.5)^d^
5.9	60	74.3	(4.08±0.04)^c^	(12.4±1.1)^d^	(0.38±0.05)^ab^	(54.9±0.1)^e^	(178.5±0.9)^h^	(27.6±0.1)^e^
13.1	20	67.63	(4.24+0.08)^c^	(15.0±0.5)^a^	(0.37±0.08)^ab^	(55.6±0.2)^e^	(278.2±1.3)^a^	(33.1±0.1)^b^
13.1	40	65.41	(4.7+0.2)^bc^	(14.0±0.0)^abcd^	(0.34±0.01)^ab^	(57.6±0.2)^d^	(262.0±0.3)^c^	(28.3±0.6)^e^
13.1	60	73.56	(4.7+0.3)^bc^	(12.9±0.2)^cd^	(0.32±0.05)^ab^	(59.9±0.9)^c^	(215.0±0.1)^g^	(27.8±0.9)^e^
19.7	20	52.25	(5.7±0.5)^a^	(15.0±1.1)^a^	(0.33±0.04)^ab^	(60.0±0.7)^c^	(275.6±0.3)^b^	(35.1±0.7)^a^
19.7	40	59.00	(5.2±0.1)^ab^	(14.7±0.0)^ab^	(0.30±0.00)^ab^	(63.2±0.1)^b^	(247.9±0.5)^d^	(31.2±0.1)^cd^
19.7	60	61.84	(4.7±0.0)^bc^	(14.3±0.7)^abc^	(0.28±0.06)^b^	(67.1±0.7)^a^	(218.4±0.2)^f^	(27.5±0.5)^e^

Results are shown as mean value±standard deviation (*N*=3). Different letters within a column indicate a significant difference based on Tukey's HSD test at p<0.05. DE=dextrose equivalent, WAI=water absorption index, WSI=water solubility index, TPC=total phenolic content, GAE=gallic acid equivalents, TMA=total monomeric antocyanins, C3G=cyanidin-3-glycoside equivalent

**Table 2:** Impact of inlet temperature (*t*_i_) and maltodextrin (MD) mass fraction on the physical and chemical properties of aronia powder produced with MD DE 19.7 at constant outlet temperature (*t*_o_=80 °C)

*t*_i_/°C	*w*(MD)/%	Process efficiency/%	w(moisture)/%	Hygroscopicity/%	WAI (*m*(solid)_centrifugate_/*m*(dry sample))/(g/100 g)	WSI (*m*(solid)_supernatant_/*m*(solid)_sample_)/%	*w*(TPC as GAE)/(mg/g)	*w*(TMA as C3G)/(mg/g)
120	20	52.25	(5.7±0.5)^a^	(15.0±0.7)^b^	(0.33±0.01)^ab^	(60.0±1.4)^cd^	(275.6±1.8)^d^	(35.1±0.6)^bc^
120	40	59.00	(5.2±0.1)^ab^	(14.7±0.1)^b^	(0.30±0.01)^bc^	(63.2±0.9)^b^	(247.9±2.0)^f^	(31.2±0.2)^de^
120	60	61.84	(4.71±0.05)^bc^	(14.3±0.9)^b^	(0.28±0.00)^c^	(67.1±0.6)^a^	(218.4±1.0)^i^	(27.5±2.1)^fg^
140	20	65.37	(4.62±0.06)^bc^	(17.5±1.0)^a^	(0.35±0.02)^a^	(56.3±0.2)^e^	(325.1±1.0)^a^	(38.4±0.1)^a^
140	40	71.02	(4.6±0.2)^bc^	(15.3±0.9)^b^	(0.30±0.01)^bc^	(62.2±1.6)^bc^	(280.7±0.5)^c^	(32.6±0.5)^cd^
140	60	72.68	(4.6±0.3)^bc^	(14.5±0.5)^b^	(0.30±0.03)^bc^	(65.8±0.1)^a^	(239.4±0.3)^g^	(29.7±0.4)^ef^
160	20	58.65	(4.3±0.2)^c^	(18.2±0.9)^a^	(0.32±0.02)^abc^	(59.8±0.5)^d^	(311.0±0.7)^b^	(37.2±0.4)^ab^
160	40	54.69	(4.5±0.1)^bc^	(16.2±0.2)^ab^	(0.32±0.02)^abc^	(62.2±0.3)^bc^	(252.6±0.0)^e^	(32.9±1.1)^cd^
160	60	55.08	(4.1±0.3)^c^	(14.9±0.7)^b^	(0.30±0.01)^bc^	(65.7±0.8)^a^	(226.2±0.1)^h^	(26.9±1.3)^g^

Results are shown as mean value±standard deviation (*N*=3). Different letters within a column indicate a significant difference based on Tukey's HSD test at p<0.05. WAI=water absorption index, WSI=water solubility index, TPC=total phenolic content, GAE=gallic acid equivalents, TMA=total monomeric antocyanins, C3G=cyanidin-3-glycoside equivalent

### Process efficiency and visual appearance of the obtained aronia powders

In the majority of investigated samples recovery was greater than 50%, while in a few of them drying efficiency was even up to 75%. According to Bhandari *et al*. (*24*), spray drying recovery greater than 50% in the cyclone is regarded as the criterion for efficient drying in laboratory dryers. Also, phenomenon such as stickiness did not occur, while wall depositions were present in several cases but in the form of thin and non-significant deposits. Therefore, regarding the above-mentioned criteria, all investigated drying and powder-producing processes can be considered as efficient.

Visual appearance is a very important criterion concerning powder application. This is especially the case with aronia powders used as natural colourants. Quek *et al*. (*25*) reported that in the case of watermelon powder, if the addition of MD exceeded 10%, powders lost their attractive red-orange colour. This was not the case with the obtained aronia powders. Visual appearances of all 15 powders were adequate. Even when 60% of MD was added, they retained attractive red-purple colour.

### Moisture content of the obtained aronia powders

Moisture content is one of the most important criteria for evaluation of powder quality. If it is inadequate, it will provoke decrease of powder stability in terms of microbiological status and loss of physical properties. Criteria for moisture content in powders for application in functional food and pharmaceutical industries are provided in numerous official documentation. According to the United States Pharmacopeia-National Formulary (USP-NF) (*26*), moisture content values lower than 5% (*m*/*m*) are considered as adequate for pharmaceutical powders, including spray-dried extracts/powders.

In the present study, all obtained and analyzed powders had moisture content lower than 5%, except in the case of powders obtained with MD DE 19.7 at mass fractions of 20 and 40% and *t*_i_=120 °C. In these two cases moisture content was above 5%, more precisely 5.7 and 5.2%, respectively (Table 2). Moisture content of all other powders ranged from 4.1 to 4.71%. As moisture content of the obtained aronia powders (except the aforementioned two samples) was below 5%, and in line with 2007 requirements of USP-NF, they can be considered adequate for use as pharmaceutical powders. This low moisture content will ensure their prolonged shelf life due to low occurrence of microbiological contamination. Also, it will enable prolonged stability of their physical properties (flowability, caking, hygroscopicity, *etc*.), which is of importance when handling and using on industrial level.

Decrease of moisture content with an increase of MD mass fraction and *t*_i_ was noticed in all investigated samples. This observation was in accordance with the observation of Abadio *et al*. (*27*) and Quek *et al*. (*25*). According to Abadio *et al*. (*27*), the water content of the feed has an effect on the final moisture content of powder produced by spray drying. Addition of MD to the feed prior to spray drying increases the total solid content and reduces the amount of water available for evaporation (*28*). According to Quek *et al*. (*25*), this means that powders with lower moisture content could be obtained by increasing the mass fractions of added MD. Shrestha *et al*. (*29*) explained the decrease of moisture content with the increase of MD by the fact that MD has the capability to interfere with sugars in the fruit powder, which is highly hygroscopic in terms of absorbing the humidity from its surroundings. Therefore, higher moisture content in the two aforesaid powders (obtained with 20 and 40% MD DE 19.7 at *t*_i_=120 °C) is probably caused by a specific chemical structure of MD DE 19.7, in terms of much higher water binding capabilities than of powders prepared with MDs of lower DE. This is in accordance with Goula and Adamopoulos (*30*), who studied the effect of the addition of different types of MD (DE 6, 12 and 21) on the properties of tomato powder. Their results showed that the MD with higher dextrose equivalent (DE) causes higher moisture content in the powder. This was explained by the chemical structure of MDs with high DE, which has numerous ramifications with hydrophilic groups, and thus can easily bind water molecules from the ambient air during powder handling after spray drying. This phenomenon has been overcome by increasing *t*_i_ for powders obtained with the addition of 20 and 40% MD DE 19.7 but at higher inlet temperatures (140 and 160 °C).

### Hygroscopicity of the obtained aronia powders

The hygroscopicity can be defined as the capacity of powder to absorb the moisture from the surroundings. According to Nadeau and Puiggali (*31*), in the case of powder for pharmaceutical and/or food applications, hygroscopicity has been related to the porosity of the powder or to the amorphous glassy state of sugars present in the foods (*32*). According to Phisut (*28*), the differences in water adsorption can be explained by the chemical structure of the applied drying agent. Namely, the phenomenon of water adsorption by carbohydrates can be attributed to formation of links between the hydrogen in water molecules and the hydroxyl groups in the amorphous regions of the substrate as well as in the surface crystalline regions.

Hygroscopicity of aronia powders obtained using MDs with DE 5.9, 13.1 and 19.7 at mass fractions 20, 40 and 60%, measured after 48 hours, ranged from 12.4 to 15.0% (Table 1). According to the obtained results, the lowest hygroscopicity was in powders obtained using MD with the lowest DE (5.9). With an increase of the DE, the increase of hygroscopicity is observed. This difference in water absorption rate can be explained by the chemical structure of the agent with high number of hydrophilic groups and its higher water binding affinity. The observed behaviour is in accordance with the observation of Tonon *et al*. (*33*) that polymerization of MD influences the hygroscopicity of the powder. According to these authors, particles of açai powder produced by using MD DE 10 had lower moisture adsorption rate than samples produced using MD DE 20. As MD DE 5.9 used in this study was less hydrolyzed than MDs DE 13.1 and 19.7, it possesses lower number of hydrophilic groups, which results in lower interaction with the hydrogen present in water molecules, and therefore, lower adsorption of water. According to the results in Table 1, the hygroscopicity of the obtained aronia powders decreases with the increase of MD mass fraction, and the lowest one was observed of the powder obtained using 60% MD DE 5.9. This observation is in accordance with Phisut’s (*28*) statement that high mass fraction of MD reduces the hygroscopicity of powders obtained using spray drying. Thus, with an increase of MD mass fraction and decrease of DE, the water absorption of aronia powders can be improved, *i.e.* hygroscopicity can be reduced. Although the decrease of hygroscopicity with an increase of MD mass fraction and decrease of DE has been noticed, the difference in hygroscopicity between each sample was not so high, approx. between 0.04 and 2.1%.

Inlet temperature affects the hygroscopicity of powders produced by spray drying. According to the results present in Table 2, at the same *t*_i_ for all three investigated temperatures, the decrease of aronia powder hygroscopicity with the increase of MD mass fraction has been noticed. On the other hand, with the increase of *t*_i_ in the case of aronia powders obtained with the addition of 20 and 40% MD DE 19.7, the hygroscopicity increased. This is in accordance with the study of Tonon *et al*. (*34*), who noticed that açai powders produced at higher *t*_i_ were more hygroscopic. According to Phisut (*28*), this is related to the water concentration gradient between the product and the surrounding air, which is greater for the less moist powder. In the powders obtained with the addition of 60% MD DE 19.7, there was no significant change of hygroscopicity with the increase of *t*_i_.

### WSI and WAI

According to Hogekamp and Schubert (*35*), ideal powder behaviour implies that the powder should wet quickly and thoroughly, sink rather than float, and disperse/dissolve without lumps. These properties of powders can be closely described using calculation of water solubility index (WSI) and water absorption index (WAI). Solubility of powders impacts their further application. Thus, powders of high WSI can be used directly as instant food products, while those of lower WSI need to be modified. It is preferable for powders that WAI is the lowest possible, contrary to WSI. WAI depends on the availability of hydrophilic groups and the gel formation capacity of the macromolecules (*36*).

The WSI of aronia powders ranged from 47.1 to 67.1% (Table 1 and Table 2). An increase of WSI with an increase of MD mass fraction was noticed when using all three MD types. This observation is in accordance with those of two research groups (*19*, *26*) that explored the production of ginger and orange juice powder. Aronia powders obtained using MD DE 19.7 had a higher WSI than powders obtained using MD DE 5.9 and 13.1. More precisely, the increase of WSI with the increase of DE at all observed MD mass fractions was noticed (Table 1). This observation can be explained by the chemical structure of each investigated MD. Since MDs with lower DE are less hydrolyzed than MDs with a higher DE, they possess a lower number of hydrophilic groups for building the links with surrounding molecules of water. According to the obtained results, it can be concluded that WSI of aronia powders can be improved by applying MDs with a higher DE and with the addition of higher mass fraction of MDs. The desired WSI was observed in aronia powder obtained by the addition of 60% MD DE 19.7, while the second best were WSI of powders obtained by the addition of 40% MD DE 19.7 at *t*_i_=120 °C, and of 60% MD DE 19.7 at *t*_i_=140 and 160 °C (Table 2). Thus, these four powders would be the best choice as ingredients in instant food products. According to the results, *t*_i_ did not show significant effect on the WSI. Similar observation was reported by Sousa *et al*. (*37*) during the investigation of spray drying of tomato powders. WAI of aronia powders ranged from 0.28 to 0.41 g per g dry powder (Table 1 and Table 2). Decrease of WAI with increase of MD mass fraction was noticed, and this was in accordance with the study of Grabowski *et al*. (*38*), who noticed that adding MD reduced the water-holding capacity of the sweet potato powders. Likewise, we observed the decrease of WAI with an increase of DE in the applied MD at all three mass fractions.

### Wettability

Wettability of powder is an important property involved in many practical problems such as: characterizing the dispersibility of a powder in liquid, evaluating the bioavailability of a medicine, selecting a liquid binder to granulate the powder, *etc*. (*39*). According to Buckton (*40*), improving the wettability of a drug leads to weaker agglomeration of drug particles in contact with the liquid. Thus, the dissolution rate of the drug powder is increased because the surface area, definitely wetted by the solvent, is greater. During the wettability measurements, it was detected that the produced aronia powders had nearly the same contact angles (*Θ*) with polar and nonpolar solvents (Table 3 and Table 4). The angle of contact with water ranged from 37.20 to 45.34, while the angle of contact with diiodomethane ranged from 29.82 to 41.13°. This means that the produced aronia powders can be well dissolved in both media, hydrophilic and lipophilic. Since in both cases contact angles were between 0 and 90°, wettability of aronia powders can be characterized as high. No significant impact of *t*_i_ on the wettability was noticed.

**Table 3:** Influence of maltodextrin (MD) type and mass fraction on wettability, polarity and particle size of the aronia powders obtained at inlet and outlet temperature of 120 and 80 °C, respectively

DE of MD	*w*(MD)/%	*Θ*/˚		*γ*/(mN/m)	Polarity/%	Particle size/μm	
Water	Diiodomethane	Length	Width
5.9	20	45.34	29.82	65.46	40.13	(6.45±1.17)^b^	(4.47±2.02)^c^
5.9	40	44.13	30.60	65.30	38.85	(7.12±2.49)^b^	(5.89±1.21)^c^
5.9	60	43.27	32.45	64.78	37.91	(7.89±2.01)^ab^	(6.01±1.57)^bc^
13.1	20	37.20	38.76	65.88	44.76	(8.06±1.04)^ab^	(6.67±0.67)^abc^
13.1	40	37.63	39.50	65.97	45.20	(8.13±1.56)^ab^	(6.11±1.23)^bc^
13.1	60	38.96	40.12	66.01	45.98	(9.53±1.13)^ab^	(7.55±2.01)^abc^
19.7	20	40.29	37.90	64.40	42.87	(9.98±1.26)^ab^	(7.63±0.98)^abc^
19.7	40	41.70	38.21	64.33	42.90	(11.72±1.21)^a^	(9.75±0.67)^ab^
19.7	60	42.98	39.72	65.87	43.78	(12.32±1.09)^a^	(10.02±0.98)^a^

Results are shown as mean value±standard deviation (*N*=3). Different letters within a column indicate a significant difference based on Tukey's HSD test at p<0.05. DE=dextrose equivalent, *Θ*=contact angle, *γ*=surface free energy

**Table 4:** Impact of inlet temperature (*t*_i_) and maltodextrin (MD) mass fraction on wettability, polarity and particle size of aronia powders produced with MD DE 19.7 at constant outlet temperature (*t*_o_=80 °C)

*t*_i_/°C	*w*(MD)/%	*Θ*/˚		*γ*/(mN/m)	Polarity/%	Particle size/μm	
Water	Diiodomethane	Length	Width
120	20	40.29	37.90	64.40	42.87	(10.0±1.3)^ab^	(7.6±0.9)^ab^
120	40	41.70	38.21	64.33	42.90	(11.7±1.2)^a^	(9.8±0.7)^a^
120	60	42.98	39.72	65.87	43.78	(12.3±1.1)^a^	(10.0±1.0)^a^
140	20	39.21	41.13	65.89	45.98	(5.0±2.1)^c^	(4.1±0.1)^b^
140	40	38.80	40.03	65.25	44.93	(5.8±1.8)^bc^	(7.1±1.7)^ab^
140	60	38.76	39.87	66.01	44.86	(5.9±1.0)^bc^	(4.0±1.9)^b^
160	20	39.92	38.34	64.36	41.45	(6.4±0.1)^bc^	(4.0±1.4)^b^
160	40	40.95	36.47	65.15	42.53	(6.7±2.4)^bc^	(4.7±1.1)^b^
160	60	42.76	37.82	65.39	43.45	(7.2±0.4)^bc^	(4.0±2.1)^b^

Results are shown as mean value±standard deviation (*N*=3). Different letters within a column indicate a significant difference based on Tukey's HSD test at p<0.05. *Θ*=contact angle, *γ*=surface free energy

Based on further results of surface free energy (*γ*) and the polarity (Table 3 and Table 4), the produced aronia powders can be considered as powders with hydrophilic character and of high polarity, which is of importance when considering their possible final application.

### Aronia powder particle size, morphology and structure

Table 3 and Table 4 summarize the average particle size of the obtained powders determined by optical microscopy. The applied spray drying procedure provided the micronized particles in all cases. The length of powder particles ranged from 5.0 to 12.32 μm, while width ranged from 4.0 to 10.02 μm. Results from Table 4 showed that there was a difference between the sizes of particles in the powders obtained with different MDs. The application of MD with the highest DE (MD DE 19.7) resulted in the production of powders with the highest values of both length and width. Also, the increase of particle length and width with the increase of MD mass fraction was noticed. Generally, higher applied *t*_i_ (140 and 160 °C) resulted in the production of particles with lower values of length and width than those obtained at *t*_i_=120 °C (Table 4), but there was no clear dependence between the decrease of particle size and the increase of inlet temperature.

**Fig. 1:**
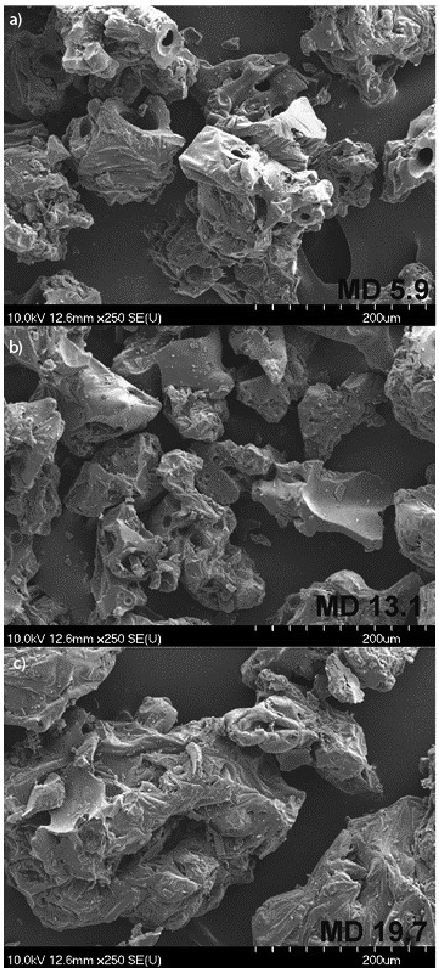
Morphology of maltodextrins (MD) with different dextrose equivalents (DE): a) 5.9, b) 13.1 and c) 19.7

Using SEM, morphologies of MDs and obtained powders were examined. Fig. 1 shows the morphologies of the applied MDs with DE 5.9, 13.1 and 19.7. The surfaces of the applied MDs were very similar, rugged with big pores. The average size of MDs was as follows: DE 5.9 (142±6) µm, DE 13.1 (165±5) µm and DE 19.7 (243±10) µm.

**Fig. 2:**
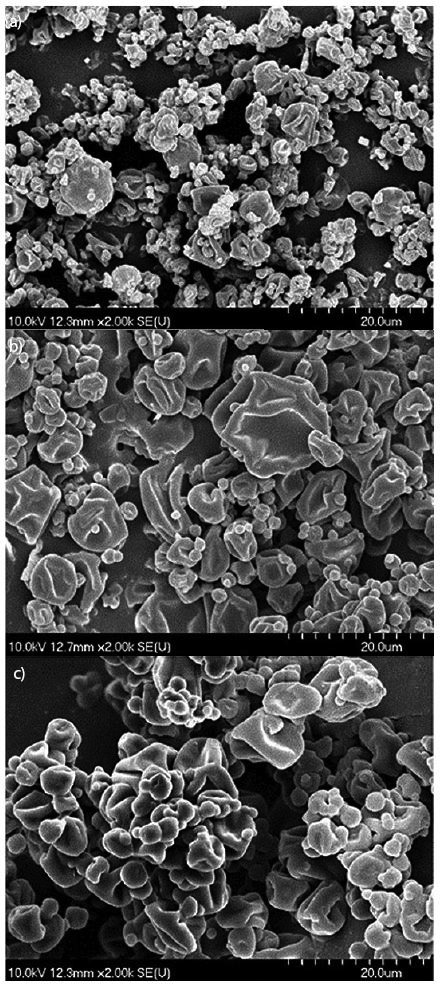
Effect of the addition of maltodextrin (MD) with different dextrose equivalents (DE) on the structure of aronia powders: a) powder obtained with the addition of MD DE 5.9, b) powder obtained with the addition of MD DE 13.1, and c) powder obtained with the addition of MD DE 19.7

**Fig. 3:**
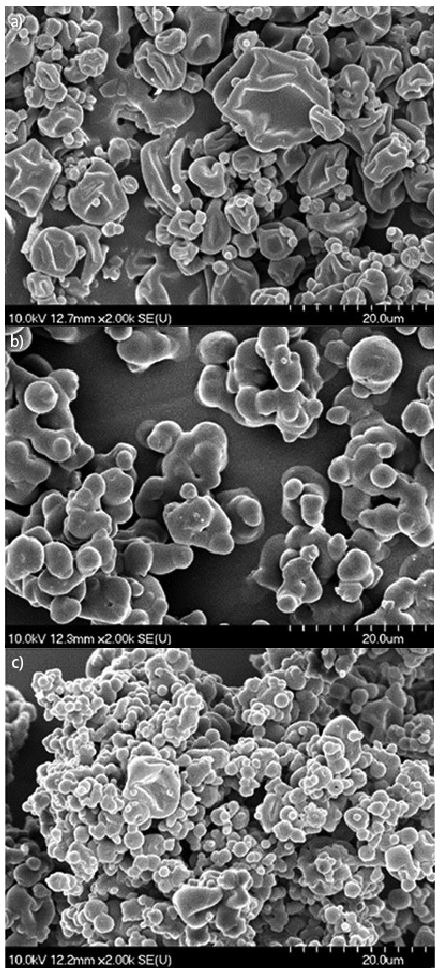
Effect of inlet temperature on the structure of aronia powders produced with 40% MD DE 19.7 obtained at: a) 120 °C, b) 140 °C, and c) 160 °C

SEM analysis of powders obtained at *t*_i_=120 °C showed that the particles of aronia powders had mainly roundish shape and micronized size. The higher MD mass fraction resulted in a slightly greater particle size. In all powders, besides very small spherical (2-3 µm) particles, greater (10-20 µm) porous particles were also found. Fig. 2 shows the morphologies of aronia powders obtained at *t*_i_=120 °C with the addition of different types of 40% MDs. This figure interprets how the morphology changes when a different type of MD is used. SEM analysis showed that the increase of *t*_i_ can help to decrease the size of powder particles. This analysis also showed that the increase of *t*_i_ can affect the production of more monodisperse distribution. Fig. 3 shows how the morphology and size of particles in powder produced with the addition of 40% MD DE 19.7 were changed by the increase of *t*_i_.

**Fig. 4:**
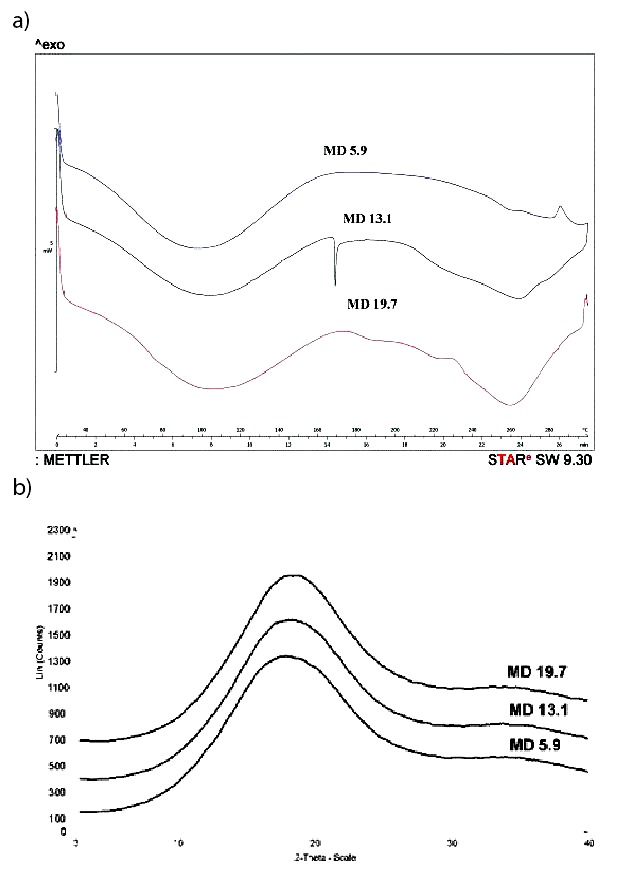
Images show: a) differential scanning microscopy curves, and b) X-ray powder diffraction patterns of maltodextrins (MD) with different dextrose equivalents (19.7, 13.1 and 5.9) used for aronia powder production

Fig. 4 shows the chemical structure of the applied MDs. According to the thermoanalytical measurements, the DSC curves (Fig. 4a) indicated the water loss of the samples close to 100 °C and a very small endothermic peak at 172 °C for MD DE 13.1. This also means that sharp endothermic peak was not present on the curves; therefore, the crystalline structure of active agents and matrix did not build up. On the XRPD patterns (Fig. 4b), characteristic peaks could not be seen; therefore, their amorphous state was verified by this method. Spray drying is an integration procedure to get final solid micronized products. All analyzed powders showed amorphous character. Thus, no energy is required to break the bonds during the dissolution process (drug release), like when it is in crystalline form. In general, bioavailability is defined as the rate and extent of drug absorption. The rapid release of a drug leads to higher absorption rates. Therefore, we can predict faster dissolution and improved efficacy.

### Analysis of health beneficial constituents of aronia powder

Horszwald *et al*. (*41*) reported that spray drying preserves higher levels of total phenolic compounds than other drying techniques. In this study, the total phenolic content (TPC) as GAE in aronia powders obtained by spray drying ranged from 178.5 to 325.1 mg/g of dry powder (Table 1 and Table 2). As such, it was significantly higher than the TPC reported in scientific literature for other aronia products. Thus, it was approx. 2- to 10-fold higher than the content reported by Oszmiański and Wojdylo (*3*) in aronia dried fruits (78.5 mg/g), aronia pomace (105.8 mg/g), and aronia juice (37.2 mg/g). It was observed that the increase of MD mass fraction decreases TPC in all MD types and at all applied *t*_i_. This is logical because total content of active material decreased. Thus, the increase of MD DE 5.9 from 20 to 60% decreases the TPC for 35.7%, while the increase of the mass fraction of MD DE 13.1 and DE 19.7 from 20 to 60% decreases TPC for 22.7 and 13.1%, respectively. In the case of the powders obtained by adding 20 and 40% MD, there was no significant difference between the TPC in powders produced with MDs with different DE. However, in the case of 60% MD, this difference was significant and it seems to decrease with the increase of DE. According to the results from Table 2, there was a significant difference among the powders obtained at different *t*_i_. The lowest TPC was measured in the powders obtained at *t*_i_=120 °C. The highest TPC was measured in aronia powders produced at 140 °C. In comparison to TPC at 140 °C, at *t*_i_=160 °C it decreased, probably due to degradation of thermosensitive phenolics.

Aronia powders showed high content of total monomeric anthocyanins expressed as cyaniding-3-glycoside equivalents ranging from 26.9 to 38.4 mg/g of powder (Table 1 and Table 2). The results were mostly in accordance with the results reported by Horszwald *et al*. (*41*). In general, total monomeric anthocyanins make approx. 11-15% of TPC in the produced aronia powders. Like TPC, total monomeric anthocyanin content decreases with the increase of MD mass fraction in powders. There was no significant difference in the total monomeric anthocyanin content in the powders obtained using different types of MDs. Therefore, it can be concluded that the type of MD DE does not affect the content of monomeric anthocyanins in aronia powders. There was no significant difference in the total monomeric anthocyanin content in the powders obtained at different *t*_i_ with 20 and 40% MD. Conversely, in the case of powders obtained with the addition of 60% MD, the difference was significant. The highest content of total monomeric anthocyanins was detected in aronia powder obtained using 20% at *t*_i_=140 °C, while the lowest was determined in the powder obtained at 160 °C using 60% MD DE 19.7.

## CONCLUSIONS

This study was performed in order to investigate the possibility of aronia fruit dust utilization and recycling through application of ultrasound-assisted extraction followed by spray drying. The results showed that thus processed aronia fruit dust can be successfully used to produce amorphous powders of adequate moisture content, with micronized roundish particles and very high mass fraction of bioactive compounds, particularly total phenols. The obtained powders were of fast dissolution rate, high water solubility index (WSI) and low water absorption index, thus these properties could enable utilization of powders as constituents of fast dissolving products, such as instant tea, instant juices, effervescent drinks, *etc.* Comprehensive analyses of all investigated powders and their characteristics (physical, structural and chemical) indicate that the powder produced at inlet temperature *t*_i_=140 °C using 40% maltodextrin (MD) with dextrose equivalent (DE) 19.7 is the one with the most appropriate properties. The study also investigated the influence of main spray drying parameters (MD type and mass fraction, and *t*_i_) on process and powder properties (chemical and structural). It was observed that an increase of MD mass fraction decreases the moisture content, powder hygroscopicity and content of bioactive compounds, while increases the WSI and particle size. Increase of MD DE increases the powder hygroscopicity and WSI, while the increase of *t*_i_ induces a decrease of moisture content in aronia powders.
